# Electrophysiological evidence for internalized representations of canonical finger-number gestures and their facilitating effects on adults’ math verification performance

**DOI:** 10.1038/s41598-021-91303-2

**Published:** 2021-06-03

**Authors:** Fabian C. G. van den Berg, Peter de Weerd, Lisa M. Jonkman

**Affiliations:** Department of Cognitive Neuroscience, Faculty of Psychology and Neuroscience, Maastricht University, Oxfordlaan 55, 6229 EV Maastricht, The Netherlands

**Keywords:** Neuroscience, Cognitive neuroscience, Learning and memory

## Abstract

Fingers facilitate number learning and arithmetic processing in early childhood. The current study investigated whether images of early-learned, culturally-typical (canonical), finger montring patterns presenting smaller (2,3,4) or larger (7,8,9) quantities still facilitate adults’ performance and neural processing in a math verification task. Twenty-eight adults verified solutions to simple addition problems that were shown in the form of canonical or non-canonical finger-number montring patterns while measuring Event Related Potentials (ERPs). Results showed more accurate and faster sum verification when sum solutions were shown by canonical (versus non-canonical) finger patterns. Canonical finger montring patterns 2–4 led to faster responses independent of whether they presented correct or incorrect sum solutions and elicited an enhanced early right-parietal P2p response, whereas canonical configurations 7–9 only facilitated performance in correct sum solution trials without evoking P2p effects. The later central-parietal P3 was enhanced to all canonical finger patterns irrespective of numerical range. These combined results provide behavioral and brain evidence for canonical cardinal finger patterns still having facilitating effects on adults’ number processing. They further suggest that finger montring configurations of numbers 2–4 have stronger internalized associations with other magnitude representations, possibly established through their mediating role in the developmental phase in which children acquire the numerical meaning of the first four number symbols.

## Introduction

The very first representations of quantities that children use are fingers, allowing them to communicate numerical information even before having acquired any other numerical representations such as number words or Arabic digits^1,2^. Learning numerical symbols is a complicated and lengthy process, as shown by reports of young children needing 1–1.5 years to learn the meaning of the first four number words^3,4^. One aspect that might make number–symbol learning so difficult is the requirement of making a transition from an analog, non-symbolic representation of numbers (e.g., several items in a set, such as three apples) to a symbolic representation^5^. It has been suggested that fingers might mediate this transition in early childhood, acting as a base or aid when memory capacity and strategy use is still immature^6–8^. The importance of fingers for number symbol learning is further indicated by findings of finger-number representations facilitating arithmetic processing^9–11^. Although there is some evidence that fingers (in the form of counting) help the early learning of number–symbol relationships^8^, little is known about the cognitive processes enabling this type of learning. Moreover, understanding how finger-number representations might facilitate symbol acquisition is important to address potential symbol learning problems in young children in a later stage. In the present study, we combined behavioral assessments with ERP measurements to investigate whether the potentially privileged role of culture-specific finger-number gestures in early numerosity learning would have left traces in the adult brain.

The underlying idea of the present study is that due to their frequent use in early number learning and daily communication, culture-specific finger-number gestures become internalized and connected to other existing or simultaneously developing non-symbolic or symbolic representations of magnitude or number, rather than only having a function as a temporary external aid during the acquisition of symbolic number knowledge. An important unexplored issue is whether the strength of internalized semantic connections between finger- and other representations of number differs between numbers smaller than five represented by one hand and numbers larger than five represented by two hands. Current models of numerical development and empirical evidence propose that only the first four number symbols are learned by mapping them directly onto their corresponding non-symbolic representation of quantity^5,12–14^. Once children master these first four symbols, they can use symbolic properties such as ordinality, to infer the numerical meaning of number symbols higher than four without referring to their corresponding non-symbolic representations^12,15^. When number gesture representations function as an initial bridge to associate abstract number symbols (e.g., number words) to earlier present non-symbolic representations of magnitude^16,17^, stronger functional links between culture-specific finger-number gestures and other representations of number may be present for the first four numbers that are assumed to be learned via direct non-symbolic–symbolic mapping.

Fingers can be used to represent quantities in different ways; one way is during counting when fingers are raised sequentially in a certain order and one finger represents one quantity. Another way in which fingers can be used to communicate and represent quantities is by holding up a hand with three raised fingers simultaneously (as a pattern) to indicate the number three, ﻿dubbed ‘montring’ by Di Luca and Pesenti^18^. Both counting and montring finger configurations are referred to as canonical because they have to be actively acquired and conform to fixed culture-specific habits. Several behavioral studies in adults have reported facilitating effects of canonical finger-number representations on the accuracy and speed of symbolic number retrieval or processing. Di Luca et al.^18,19^ demonstrated that the naming of the number represented by early-learned canonical (counting and montring) finger configurations was faster and more accurate than in the case of unfamiliar, non-canonical finger configurations. In a follow-up study, the same authors demonstrated that priming by canonical finger-number montring configurations facilitated the subsequent recognition of other symbolic representations such as Arabic digits in the single-hand range^1–5,20^. These facilitating effects of canonical finger-number configurations have also been shown to extend to arithmetical operations in a study by Badets et al.^21^. In this study, adult participants were presented with a math verification task in which a simple math-sum was given (e.g., 3 + 4), followed by a sum solution that had to be verified as correct or incorrect. Sum solutions were presented in the form of finger-number configurations or as a number of rods. Participants verified sum solutions faster and more accurately when presented in the form of a canonical finger pattern rather than rods, indicating that arithmetic was, to some extent, still embodied in adults. Thus, multiple behavioral studies show facilitating effects of canonical finger-number representations on symbolic number processing in adults, supporting the idea of shared internalized cognitive processes between canonical finger- and other symbolic number representations.

Various authors have speculated on the processes underlying the number processing benefits of canonical finger montring configurations and suggested that, at least in adults with fully developed symbolic number systems, the early-learned canonical finger configurations have acquired a special status in long-term memory^20,22,23^. Specifically, due to the frequent use of fingers during the developmental phase in which children first acquire early numeric and math skills^7,24,25^, strong semantic associations may have formed between finger-representations of numbers and other non-symbolic (analog quantities) and symbolic (number words, Arabic digits) number representations. As a result, canonical finger representations of numbers may facilitate numeric processes, including number recognition and retrieval. By contrast, the non-familiarity of non-canonical finger-number configurations and the absence of associations with other types of symbolic and non-symbolic number representations, renders number processing more time-consuming and error-prone^18–21^.

However, behavioral measures do not permit the identification of the brain processes responsible for the above reviewed facilitating effects of canonical finger-number configurations on symbolic number processing. Event-related brain potentials can provide information about semantic processing differences between canonical and non-canonical finger-number gestures in the brain. Prior number-processing ERP studies with standard symbolic number stimuli (e.g., Arabic digits) have consistently found that a positivity above the right temporal-occipito-parietal hemisphere peaking around 200–250 ms after stimulus presentation, called the P2p (e.g. the second posterior positivity) by Dehaene^26^, is the first ERP component modulated by numerical semantic processing demands in tasks requiring magnitude judgements^26–29^. These studies all used so-called symbolic number comparison tasks in which one has to decide whether a presented number symbol (digit) is larger or smaller in magnitude than another internal reference number, which requires retrieval of the symbols’ associated magnitude information (its number semantics) and comparison of the magnitude of the presented symbol with that of the internal reference number. The modulation of the amplitude of the P2p by this magnitude comparison process, and more specifically by the numerical distance between two to-be-compared number stimuli on the mental number line, has led multiple authors to conclude that the P2p in symbolic numerical processing tasks, reflects the processing stage in which one has first access to semantic (analog) quantity information associated with the number symbols^26–29^. In the majority of the above number symbol comparison studies, the P2p numerical distance effects were larger above the right than left posterior hemisphere and Dehaene^26^ accordingly source-localized the response-locked P2p distance effect in the right parieto-occipito-temporal junction (but see Pinel^27^, who localized the P2p distance effect in bilateral parietal cortex). Further evidence for a functional link between posterior right-lateralized P2p activity and activation of/or access to quantity representations comes from a recent number symbol learning study by van den Berg et al.^13^. In this study, only novel symbols that had acquired numerical meaning by successfully associating them with their corresponding non-symbolic (quantity) representation elicited a P2p response. Interestingly, two magneto-encephalography (MEG) studies investigated the spatiotemporal processing pattern accompanying the extraction of the meaning or connotation of familiar (versus unfamiliar) hand gestures, for example gestures for ‘stop’ or ‘ok’^30^, or natural versus distorted finger postures^31^. Both studies reported enhanced activity in a 200–380 ms time window, either in bilateral extra-striate cortex^31^ or in right-hemisphere inferior occipito-temporal, inferior parietal and right STS regions^30^, similar to the timing and location of the P2p in above discussed number symbol processing studies.

A second endogenous ERP component that has consistently been reported to be modulated in tasks requiring the retrieval and comparison of magnitude codes associated with symbolic and non-symbolic number stimuli, is the centro-parietal P3 that occurs between approximately 300–500 ms after stimulus presentation^26,28,29,32,33^. Although the literature is less consistent on the functional interpretation of magnitude comparison (e.g., numerical distance) effects on the P3, based on its resemblance to the classical centro-parietal P3^34^, it has been linked to later stimulus evaluation/categorization processes, with higher amplitudes reflecting easier categorization and/or higher response confidence^26,29^, likely due to better memory representations.

To the best of our knowledge, only two recent studies investigated ERP responses to canonical finger-number stimuli using different symbolic number tasks. Proverbio and Carminati^35^ presented adult participants with a math verification task in which sum solutions shown as Arabic numerals had to be verified. Arabic numerals were flanked by to-be-ignored, task-irrelevant, canonical finger-counting configurations of numbers 0–10 showing the same (congruent) or a different (incongruent) number than indicated by the Arabic numeral. Results showed that congruent finger-number configurations interfered with the processing of the Arabic numeral, yielding lower accuracy, which is in contrast with earlier reported facilitating effects of canonical finger-number patterns on math verification^21^. Proverbio and Carminati (2019) focused their ERP analyses on frontal and central electrodes and did thus not report on effects of finger-stimuli on posterior P2p or P3 components related to semantic processing of number stimuli in the prior number processing literature. Furthermore, the study only included canonical (not non-canonical) finger–number stimuli and the task did not require explicit processing of the numerical meaning of the finger patterns. In another study by Soylu et al.^35^, ERPs were measured in response to single hand stimuli representing numbers 1–4 showing counting, montring, and non-canonical finger-number configurations. The participant’s task was to decide whether the quantity shown by the fingers was the same or different from a later appearing Arabic digit, requiring active retrieval of the number represented by the fingers. The authors reported that compared to both counting and non-canonical finger configurations, montring configurations were processed faster and more accurately which went along with enhanced early perceptual P1/N1 responses. The amplitude of the later endogenous centro-parietal P3 component, measured in a 250–500 ms time window, was however enhanced to the same extent by both canonical (counting and montring) finger stimuli. Based on these findings, the authors concluded that only finger montring patterns showed facilitated processing and elicited higher early perceptual/attentional processing (as indexed by P1 and N1), whereas, in line with earlier suggestions by Di Luca et al.^20^, the higher P3 response to both canonical (counting and montring) stimuli was interpreted as signifying their retrieval from memory. These findings suggest that canonical finger-number montring patterns are easier to enumerate because of the faster automatic activation/retrieval of their associated numeric representations from long-term memory.

These above discussed studies were either limited to studying canonical finger-number representations of smaller numbers 1–4^20,22^ or did use a larger range ﻿up to 10^35^, but did not include numerical range as a factor in their analyses. As explained above, making such a distinction is important because of propositions that fingers may scaffold number symbol acquisition by facilitating the process of mapping abstract number symbols (digits, words) onto non-symbolic representations of magnitude. Because only the first four number symbols are suggested to be learned via such one-to-one symbolic—non-symbolic mapping^5,12–14^, associations between canonical finger-montring patterns and other representations of number might be stronger for the small (1–4) than for the larger (6–9) number range.

The aim of the present study was two-fold: (1) to investigate the semantic processing of canonical finger-number montring gestures (versus non-canonical gestures) and their facilitating effects on number magnitude comparison in the adult brain by, besides on behavior, also investigating effects on the P2p and P3-ERP components that have in earlier studies been linked to early and late stages of number semantic processing (see above) and (2) to compare the semantic processing of canonical finger gestures showing small numbers 2–4 represented on one hand with those showing larger numbers 7–9 represented on two hands. To investigate this, we used a simple math verification task ﻿based on^21,35^, in which participants were required to compute a sum by adding two visually presented one-digit numbers, and had to verify whether a subsequently presented canonical or non-canonical finger-number pattern (numerical ranges 2–4 or 7–9) showed the correct or incorrect sum solution. With respect to the first aim, we expect that if canonical finger-number gestures are indeed stored in long-term memory and have gained associations with other numeric (quantity) representations, we will find a main effect of canonicity on the amplitude of the right-lateralized P2p component^26,28,29^. More specifically, we hypothesize that canonical finger-number gestures will elicit faster math verification responses and higher (right-lateralized) P2p amplitudes than non-canonical finger-number gestures (canonicity × hemisphere interaction). We further expect that canonical finger-number gestures will elicit higher amplitudes of the later centro-parietal P3-ERP component than non-canonical finger-number gestures (main canonicity effect), evidencing their easier, or less ambiguous classification due to their storage in and retrieval from long term memory^22,36^. With respect to the second aim; if small canonical finger-number gestures (2–4) have developed stronger semantic numeric associations than canonical finger patterns gesturing larger numbers 7–9, we expect to find canonicity × numerical range interaction effects on reaction time, P2p (including factor hemisphere) and P3 amplitude, with stronger canonicity effects (faster comparison reaction time, higher right-posterior P2p amplitude and higher centro-parietal P3 amplitude to canonical than non-canonical) for finger gestures showing small sum solutions (2–4) than for gestures showing larger sum solutions (7–9). Finally, in math verification paradigms correct sum solution trials standardly elicit better performance and higher P3 amplitude than trials showing an incorrect sum solution^35,36^. To be able to distinguish between facilitating effects of canonicity and numerical range of the finger patterns and effects of presented sum solutions being correct or incorrect, all above analyses will also include the trial-type factor correct/incorrect sum solutions.

## Results

All analyses included within-subject factors Canonicity (2 levels: canonical and non-nanonical), sum Solution (2 levels: correct and incorrect sum solutions), and numerical Range (2 levels: low [numbers 2, 3, 4] and high [numbers 7, 8, 9]). Only the P2p amplitude analysis included an extra factor Hemisphere (2 levels: left and right).

### Behavioral results

#### RT

**Figure 1:**
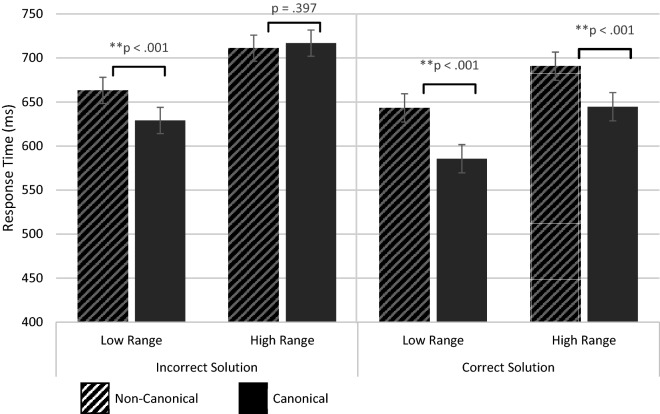
Canonicity effects on reaction time for the low and high numerical ranges and for incorrect and correct sum solution trials (three-way Canonicity × Numerical Range × Solution interaction).

The analysis yielded significant main effects of Canonicity (F[1,28.86] = 79.50; *p* < 0.001), Solution (F[1,51.96] = 51.59; *p* < 0.001), and Range (F[1,24.98] = 97.23; *p* < 0.001), and a three-way interaction between these factors (F[1,37.26] = 6.29; *p* = 0.017: see Fig. 1 and Table 1). The three-way interaction was further explored by splitting up on the Solution factor. For correct sum solution trials the Canonicity × Range interaction was not significant (F[1,24.40] = 2.09; *p* = 0.161), but main effects of Canonicity (M_Diff_ = − 51.88 ms; t(31.27)N_Can–Can_ = 9.93, *p* < 0.001) and Range (M_Diff_ = 53.18 ms; t(24.84)_High–Low_ = 6.16, *p* < 0.001) respectively showed that sum verification time was significantly faster for canonical than non-canonical finger patterns (for both patterns 2–4 and 7–9) and decisions were overall faster for small than large sum solutions (irrespective of their canonicity). For incorrect sum solution trials, a significant Canonicity × Range interaction was found (F[1,32.51] = 22.08; *p* < 0.001), and further (Bonferroni corrected) pairwise comparisons showed a significant canonicity effect only for finger patterns showing numbers 2–4 (faster verification times for canonical; M_Diff_ = 34.10 ms; t(50.23)N_Can–Can_ = 5.05, *p* < 0.001), whereas the canonicity effect for finger patterns 7–9 was non-significant (M_Diff_ = − 5.74 ms; t(50.23)N_Can–Can_ = − 0.85, *p* = 0.397). Comparing the range effect per canonicity-level showed a larger range effect (slower responses for the high range) for Incorrect Canonical (M_diff_ = 87.78 ms; t(49.69)_High–Low_ = 11.24, *p* < 0.001) compared to Incorrect Non-Canonical (M_diff_ = 47.94 ms; t(49.69)_High–Low_ = 6.14, *p* < 0.001) trials. These further tests thus indicate that the lack of a canonicity effect for finger patterns 7–9 on incorrect trials is due to a relatively larger increase in reaction time for canonical finger patterns 7–9 signifying incorrect solutions.

**Table 1:** Means and Standard Errors (between brackets) for behavioral and ERP data in the four Canonicity-Range conditions separated for Solution Correctness and hemisphere.

			Non-Canonical	Canonical		
Response times (milliseconds; ms)	Correct solution	Low range	643.27 (16.03)	585.53 (16.03)		
		High range	690.59 (16.03)	644.58 (16.07)		
	Incorrect solution	Low range	663.13 (14.89)	629.03 (14.89)		
		High range	711.07 (14.89)	716.82 (14.89)		
Accuracy (%)	Correct solution	Low range	78.47% (1.83%)	77.58% (1.33%)		
		High range	71.41% (2.96%)	74.60% (2.28%)		
	Incorrect solution	Low range	76.39% (2.01%)	75.69% (1.81%)		
		High Range	69.05% (2.88%)	70.04% (2.92%)		
P3 µV (Average of CP and CPz; 280–550 ms)	Correct solution	Low range	3.66 (0.32)	4.23 (0.32)		
		High range	2.94 (0.32)	3.25 (0.32)		
	Incorrect solution	Low range	2.83 (0.32)	3.44 (0.32)		
		High range	2.56 (0.33)	2.91 (0.32)		

			Non-Canonical		Canonical	
			Left	Right	Left	Right
P2p µV (P3 & P4) (220–310 ms)	Correct solution	Low range	2.42 (0.47)	3.71 (0.47)	2.26 (0.47)	3.89 (0.47)
		High range	1.99 (0.47)	2.56 (0.47)	1.95 (0.47)	2.26 (0.47)
	Incorrect solution	Low range	1.90 (0.47)	3.33 (0.47)	2.28 (0.47)	4.05 (0.47)
		High range	1.92 (0.47)	2.63 (0.47)	2.42 (0.47)	2.87 (0.47)

Values shown are estimated marginal means with Standard Errors. | Low Range = average of 2, 3 and 4; High Range = average of 7, 8 and 9.

#### Accuracy

The analysis of accuracy only showed significant main effects for Canonicity (X^2^[1] = 9.07; *p* = 0.003) and Range (X^2^[1] = 6.16; *p* = 0.013; see Table 1), none of the other terms reached significance (all *p*-values > 0.225). Accuracy was higher on canonical than non-canonical trials (M_diff_ = 2.75%; t(1)_Can–Ncan_ = 3.10; *p* = 0.002) and for finger-number configurations in the low (2–4) compared to the higher (7–9) numerical range (M_diff_ = 5.78%; t(1)_Low–High_ = 2.56; *p* = 0.010).

### ERP results

#### Posterior positivity between 220 and 310 ms (P2p)

**Figure 2:**
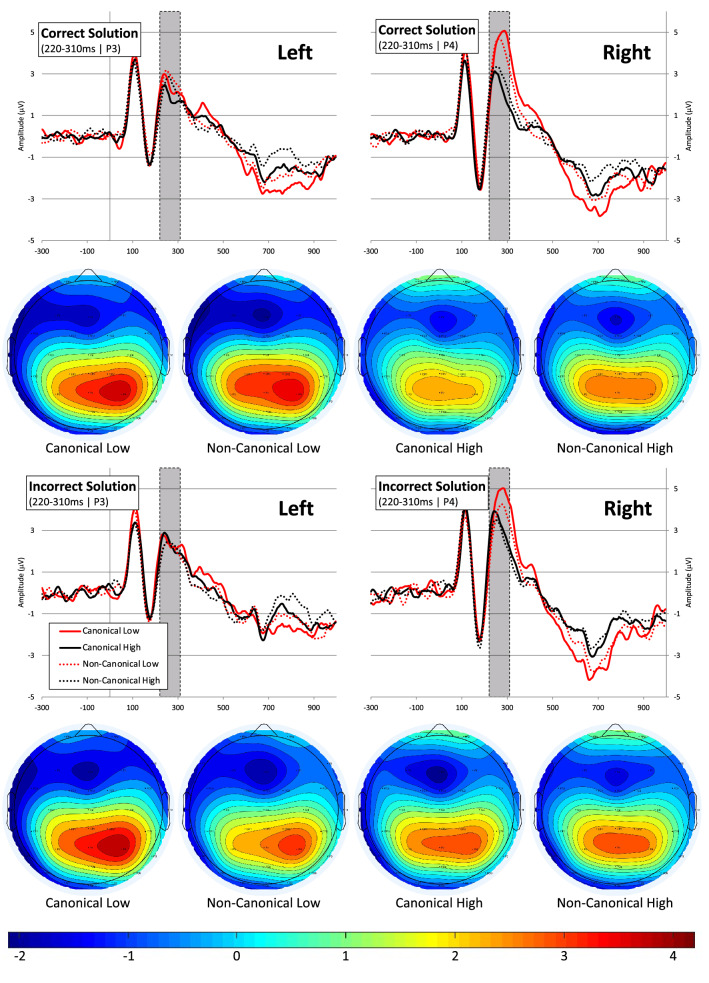
Grand average ERP’s at the left parietal (P3) electrode (left panel) and the right parietal (P4) electrode (right panel) with the P2p time window used in the analysis indicated by the grey bar. Topography plots for the 220–310 ms (P2p) time-window are shown separately for correct (top panel) and incorrect (bottom panel) sum solution trials in the four Canonicity-Range categories: Canonical-Low, Non-Canonical-Low, Canonical-High, Non-Canonical-High. The ERP plots show the grand average ERPs and topo-plots in all conditions of the experiment for purposes of evaluation of ERP quality and choice of windows and electrodes for analyses (see: Keil et al.^37^). For visualization of the specific interaction effects found on P2p and P3 amplitude we refer the reader to Fig. 4A. Images are created by the first author using ERPlab software^38^.

The grand average ERPs and topomaps presenting P2p effects are shown in Fig. 2. The analysis of the first positivity (P2p) at left and right parietal electrodes showed main effects of Canonicity (F[1,36.67] = 4.80; *p* = 0.035), Range (F[1,53.61] = 40.11; *p* < 0.001), and Hemisphere (F[1,26.77] = 5.34; *p* = 0.029). Additionally two-way interactions between Canonicity × Solution (F[1,59.02] = 10.68; *p* = 0.002) and Range × Solution (F[1,59.94] = 5.92; *p* = 0.018) were found, showing a canonicity effect (larger amplitude for canonical trials) on trials where fingers showed incorrect sum solutions (M_Diff_ = 0.46 µV; t(72.01)_Can–Ncan_ = 3.85, *p* < 0.001), but not when they showed correct sum solutions (M_Diff_ = 0.08 µV; t(72.01)_Can–Ncan_ = 0.70, *p* = 0.485). The range effect (larger amplitude for the low range) was larger for correct sum solution trials (M_Diff_ = 0.88 µV; t(111.42)_Low–High_ = 6.34, *p* < 0.001) than for incorrect sum solution trials (M_Diff_ = 0.43 µV; t(111.42)_Can–Ncan_ = 3.08, *p* = 0.003). Finally, the analyses yielded a two-way Range × Hemisphere (F[1,53.40] = 22.91; *p* < 0.001), and a three-way Canonicity, Range × Hemisphere interaction (F[1,55.06] = 4.93; *p* = 0.031). The three-way interaction was further explored by splitting on the Hemisphere factor. A significant Canonicity × Range interaction was found at P4 above the right parietal cortex (F[1,43.24] = 4.43; *p* = 0.041), but the interaction was strongly non-significant at the P3 electrode above left parietal cortex (F[1,44.70] = 0.22; *p* = 0.639; see top panel Fig. 4A). Further (Bonferroni corrected) pairwise comparisons at the right hemisphere (P4) electrode showed a significant canonicity effect signified by higher P2p amplitudes to canonical than non-canonical hands for the low (2–4) number range (M_Diff_ = 0.45 µV; t(77.07)_Can–Ncan_ = 2.75, *p* = 0.007), whereas the canonicity effect for the high range was highly non-significant (M_Diff_ = − 0.02 µV; t(77.07)_Can–Ncan_ = − 0.10, *p* = 0.919). As was expected on the basis of the non-significant interaction, no significant canonicity effects on ERP amplitude were found above the left parietal hemisphere (electrode P3) in low or high number ranges (Low: M_Diff_ = 0.11 µV; t(81.65)_Can–Ncan_ = 0.79, *p* = 0.431; High: M_Diff_ = 0.20 µV; t(81.65)_Can–Ncan_ = 1.46, *p* = 0.147).

#### Positivity between 280 and 550 ms (P3)

**Figure 3:**
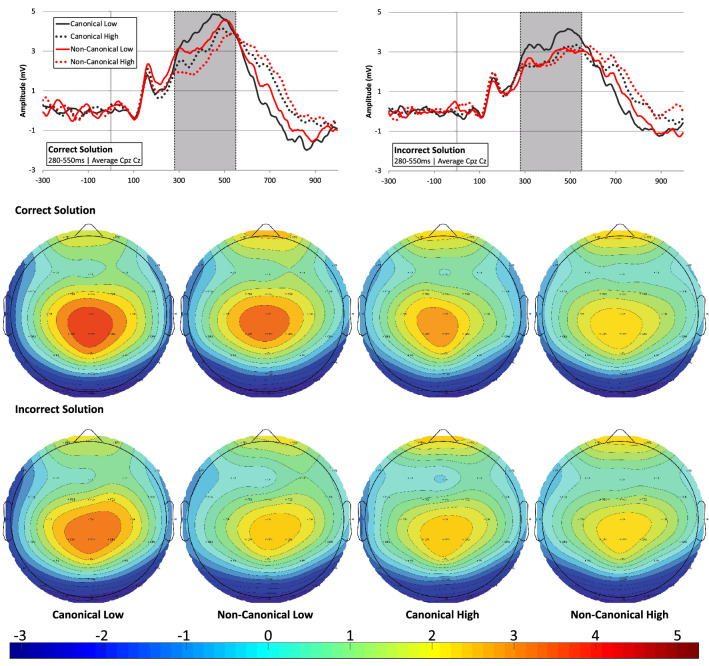
Grand average ERP’s (averaged over CPz and Cz electrodes) with the P3 time window indicated by the grey bar; left and right ERP graphs respectively show the grand average ERPs on correct and incorrect sum solution trials. Topography plots for the 280–550 ms (P3) time-window for correct and incorrect sum solution trials in the four Canonicity-Range categories: Canonical-Low, Non-Canonical-Low, Canonical-High, Non-Canonical-High. The ERP plots show the grand average ERPs and topo-plots in all conditions of the experiment for purposes of evaluation of ERP quality and choice of windows and electrodes for analyses (see: Keil et al.^37^). For visualization of the specific interaction effects found on P2p and P3 amplitude we refer the reader to Fig. 4B. Images were created by the first author using ERPlab software^38^.

**Figure 4:**
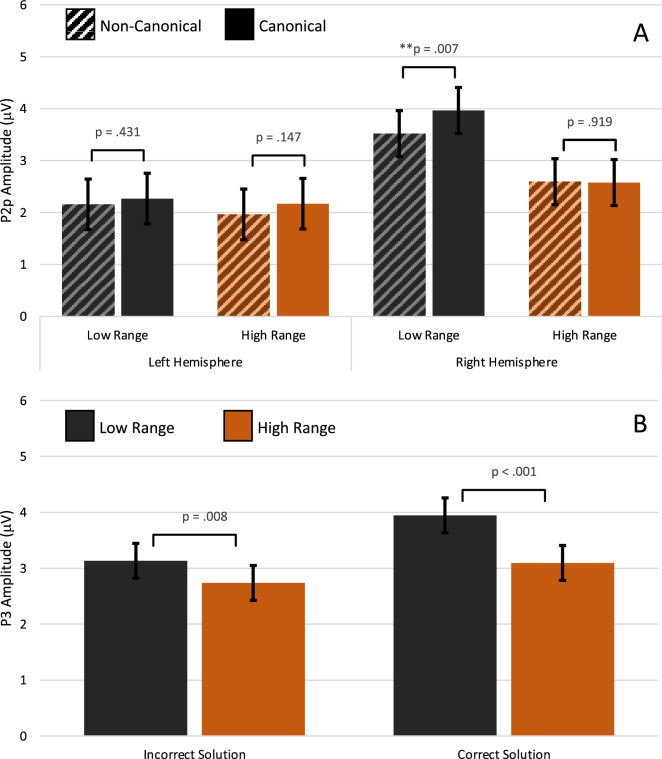
Top: Mean P2p amplitude (averaged across the 220–310 ms time window) at left and right hemisphere parietal (P3 and P4) electrodes in the four Canonicity-Range conditions: Canonical-Low, Non-Canonical-Low, Canonical-High, Non-Canonical-High. The figure shows the three-way interaction between Canonicity, Range and Hemisphere with a statistically significant canonicity effect on P2p amplitude occurring only in response to finger-number configurations showing small numerosities 2–4 above the right parietal hemisphere. Bottom: Mean P3 amplitude averaged across CPz and Cz electrodes in the four Solution-Range categories: Incorrect-Low, Correct-Low, Incorrect-High, Correct-High, showing the Solution × Range interaction with a larger numerical range effect on P3 amplitude (enhanced activation in response to hands showing small numbers 2–4) on trials with correct compared to incorrect sum solutions.

The grand average ERPs and topomaps presenting P3 effects are shown in Fig. 3. In this time window, the topography maps show a clear positivity (P3) with maximum amplitude above midline Cz and CPz electrodes and the P3 amplitude averaged across Cz and CPz was entered in the analysis. The analysis yielded main effects for Canonicity (F[1,181.99] = 19.86; *p* < 0.001), Range (F[1, 181.99] = 36.19; *p* < 0.001), and Solution (F[1, 181.99] = 32.01; *p* < 0.001), and a two-way Range × Solution interaction (F[1, 181.99] = 4.82; *p* = 0.029). Amplitude was significantly larger for Canonical finger-number representations (M_Diff_ = 0.46 µV; t(118.99)_Can–Ncan_ = 4.46). Similarly, correct solutions elicited higher P3 amplitude compared to incorrect solutions, but this difference was larger in the lower numerical range (M_diff_ = 0.81 µV; t(181,98)_Correct–Incorrect_ = 5.57, *p* < 0.001; see bottom panel Fig. 4B) than the higher range (M_diff_ = 0.36 µV; t(181,99)_Correct–Incorrect_ = 2.44, *p* = 0.016).

### Correlations between ERPs (P2p and P3) and reaction time

To investigate possible relations between the canonicity effects on reaction time and P2p and P3 amplitude, we performed two exploratory post-hoc correlation analyses: one for the P2p and one for the P3. The α-level corrected for multiple comparisons is *p* < 0.025 for the P2p analysis and p < 0.0125 for the P3 correlation analyses. Because the canonicity effect on the P2p was only significant at the right parietal electrode and only for finger montring patterns showing small numbers 2–4, a zero-order correlation was computed between right-parietal P2p amplitude and RT in response to canonical finger montring patterns 2–4 (averaged over correct and incorrect sum solution trials because for patterns 2–4 canonicity effects on P2p and RT were present in both types of trials). The zero order correlation between right-parietal P2p amplitude and RT in response to canonical finger patterns showing numbers 2–4 was not significant (*r*[26] = − 0.15, *p* = 0.45), and this did not change after controlling for right-parietal P2p amplitude and reaction time in response to non-canonical finger patterns 2–4 (*r*_*partial*_ [24] = 0.18, *p* = 0.38).

Next, similar zero-order and partial correlations (the latter controlling for P3 and RT responses to non-canonical finger patterns) were computed between mean P3 amplitude and mean RT averaged across all 6 (2–4 and 7–9) canonical finger montring patterns. This was based on the finding of a main canonicity effect on P3 (and no interaction with numerical range). Correct sum solution trials did show a significant zero-order correlation between P3 amplitude and RT in response to canonical hands (*r*[26] = − 0.48, *p* = 0.010), with higher P3 amplitude in response to canonical hands being related to faster math verification responses. Partial correlations showed that the correlation remained significant (nearing significance with corrected α-level of 0.0125) after controlling for non-canonical P3 amplitude and RT to all six configurations (*r*_*partial*_[24] = − 0.46, p = 0.018). On incorrect sum solution trials the zero-order correlation between P3 amplitude and reaction time in response to canonical finger patterns was not significant (*r* [26] = − 0.30, *p* = 0.12) and became strongly non-significant after partialling out the effect of non-canonical P3 amplitude and RT (*r*_*partial*_[24] = − 0.03, *p* = 0.87). These correlation data suggest that there is a unique relation between P3 amplitude and reaction time only for canonical finger patterns and only when finger pattern and mentally computed sum were numerosity congruent (i.e., when finger montring patterns showed the correct sum solution). But based on the post-hoc and exploratory nature of these correlation analyses and the relatively small sample size these results should be interpreted with caution and need replication in a larger study sample.

## Discussion

The present study investigated two main hypotheses: (1) whether culture-specific (canonical) finger-number gestures (versus non-canonical gestures) facilitate magnitude comparison performance in a math verification task and whether this is related to facilitated semantic processing in the adult brain (the latter evidenced by higher amplitude of P2p and P3 amplitude components that have in earlier studies been linked to early and late stages of number semantic processing (see introduction) and (2) to investigate if canonical finger gestures showing small numbers 2–4 show stronger facilitation of math verification performance due to better semantic (memory) representation than canonical gestures showing larger numbers 7–9, as evidenced by faster comparison times and stronger P2p and P3 responses.

The behavioral data showed a statistically significant interaction effect between numerical range, canonicity, and sum solution for reaction time. Further testing of this interaction showed that on trials in which finger patterns presented the correct sum solution (i.e., in which the magnitude of the computed sum and that indicated by the finger patterns was the same), both canonical small (2–4) and larger (7–9) finger patterns elicited faster magnitude comparison times than their non-canonical counterparts (i.e., a main canonicity effect was found for correct sum solution trials). These results replicate earlier reported facilitating effects of canonical finger-number configurations representing correct sum solutions on adults’ reaction time in a math verification task^21^. However, in this earlier study, canonical finger-counting patterns were not compared with non-canonical finger patterns but with quantity conveying non-finger stimuli (number of rods). Other prior studies that did compare effects of cardinal canonical and non-canonical finger patterns also reported similar facilitation effects of canonical fingers patterns 1–5 on reaction times, but in other number processing tasks^18–20,22^. Di Luca et al.^18,19^ concluded that these processing benefits of cardinal canonical finger configurations of numbers 1–5 are due to their storage in long-term memory, allowing for faster automatic recognition and retrieval of the numerosity they represent, whereas non-canonical finger patterns need elaboration (e.g., counting) to extract their numeric code. Our results add to these earlier behavioral findings, by making a distinction between canonical (and non-canonical) finger gestures showing small (2–4) or larger (7–9) quantities. Canonical finger-based representations of numbers 7–9 overall needed more processing time than those of numbers 2–4, most likely caused by the need to add up the two numbers displayed by the two hands. Nevertheless, both canonical finger-based representations of numbers 2–4 and 7–9 led to faster math verification responses on trials in which they presented the correct sum solution (i.e., in trials in which the to-be-compared stimuli were numerosity congruent).

The three-way interaction further showed that on trials in which finger patterns showed incorrect sum solutions (i.e., on numerosity incongruent trials), number-finger gestures 2–4 still facilitated comparison time compared to their non-canonical counterparts. However, the canonical finger patterns showing larger numbers 7–9 did no longer facilitate magnitude comparison (compared to non-canonical patterns 7–9) on trials where finger patterns showed an incorrect sum solution. A look at the mean reaction times shows that the abolishment of these processing benefits for canonical finger gestures 7–9 on incorrect sum solution trials is caused by a specific increase in reaction time for canonical patterns 7–9 on incorrect solution trials. Although speculative, this increase might be due to effects of numerical distance that are only present on incorrect sum solution trials where the mentally computed sum always differed with a numerical distance of 1 or 2 from the sum solution shown by the hands. A possible reason for distance effects only causing an increase in reaction time for incorrect canonical finger patterns 7–9 and not 2–4, might be that the latter have acquired stable, exact, one-to-one mapping onto spatial magnitude representations (i.e., the mental number line) causing them to be less susceptible to distortions caused by activation of adjacent numbers. In their priming study, Di Luca et al.^20^ confirmed exact mapping of canonical (and not non-canonical) finger patterns representing numbers 1–5 onto analog magnitude representations of number (e.g., number line). Such exact mapping was shown by the finding that canonical finger gesture primes showed strongest activation of target numbers to which they were close on the mental number line, with the extent of activation decreasing with an increase in the numeric distance between primes (the canonical finger patterns) and targets (Arabic digits or verbal numerals). Similar linear distance effects were not found for non-canonical finger patterns. Concluding, both small and larger canonical finger patterns benefitted magnitude comparison in the math verification task in numerosity congruent trials, but only canonical finger gestures 2–4 maintained the same processing benefits in more difficult numerosity incongruent trials where one had to make the decision that finger pattern and mentally computed sum did not show the same numerosity. As hypothesized in the introduction canonical finger patterns of small numbers 1–4 might have developed stronger (more stable) connections with other representations of magnitude due to the mediating/facilitating role that they play in early childhood when children learn to count and acquire the numeric meaning of the first four number symbols^3,4^. Recent models and findings suggest that especially the first four number symbols gain numeric meaning by mapping them with one-to-one correspondence on their earlier present, analog, non-symbolic representations of magnitude^5,12–14^, and fingers may scaffold this difficult process of connecting abstract codes of number such as number words to their corresponding magnitude in the outside world^16,17^.

The current study also measured ERPs, to gain more insight into the semantic processes underlying these facilitating effects of canonical finger-number representations on number symbol processing in the math verification task. In the early P2p time window from 220 to 310 ms, a significant three-way interaction occurred between canonicity, numerical range, and hemisphere. A significantly enhanced positivity (P2p) was present above the right (but not left) parietal cortex in response to canonical (compared to non-canonical) finger–number montring configurations, but only for those in the low (2–4) numerical range, partially corroborating the stronger canonicity effect for finger patterns 2–4 than 7–9 on reaction time that was present for incorrect but not correct sum solution trials. P2p amplitude was only enhanced in response to canonical finger patterns 2–4 (and not patterns 7–9), and this P2p response occurred independent of whether canonical finger patterns 2–4 matched or did not match with the mentally computed sum. As explained before, number comparison reaction time findings did however differ for correct and incorrect sum solution trials; on correct sum trials reaction time was faster for both canonical patterns 2–4 and 7–9. This can be explained by the fact that reaction times reflect the outcome of all previous information processing stages from early perception, early semantic processing to later more conscious memory and decision-making processes and these behavioral results are thus influenced by the (in)congruency of sum solutions. The P2p however represents a very early processing stage of automatic access to number semantics that is not affected by decision making processes, and such early numerical access is thus only shown for canonical patterns 2–4. The exploratory correlation analyses show no correlation between canonicity effects on P2p amplitude and reaction time, whereas this is the case for the later P3, leading to the tentative conclusion that the facilitating effects of canonical finger patterns on RT are likely due to facilitated processing during later semantic processing stages (see below for further discussion). No significant canonicity effects on the left- or right parietal P2p component were present for finger montring postures showing numbers 7–9. As discussed in the introduction, a previous MEG study by Nakamura et al.^30^ found similarly enhanced activation in posterior areas to meaningful versus meaningless (non-numeric) hand gestures within approximately the same time-interval and with a similar right-hemisphere dominance as the current P2p effect. The authors concluded that whereas the left hemisphere is more involved in early visual-motor processing of the hand stimuli, the right hemisphere is involved in semantic processing of the cultural meaningful hand gestures. Accordingly, the current higher right-lateralized P2p response to canonical finger-number patterns 2–4 might be a sign of the automatic activation of number semantics, more specifically the activation of associated magnitude representations in long-term memory.

Whereas not including finger stimuli, several prior ERP, fMRI, and behavioral studies provide support for a conclusion of the current right-lateralized P2p effect reflecting activation of magnitude representations associated with canonical finger patterns of numbers smaller than five. First, as reviewed in the introduction, multiple prior number processing ERP studies provide evidence for a link between the P2p and access to (spatial) representations of magnitude (e.g., on a mental number line). In a study by Dehaene et al.^26^, adult participants performed a symbolic number comparison task requiring magnitude comparisons between digits 1–9, which requires activation/retrieval of a digit’s magnitude code. A right-lateralized ERP component, similar to the present P2p in timing and location, called the P2p, was modulated by the numerical distance between the to-be-compared numbers, and was hence concluded to represent access to magnitude information. Further, in a recent ERP study by van den Berg et al.^13^ young adults learned the numerical meaning of novel symbols representing numbers 1–9 by mapping them onto corresponding quantity representations, mimicking the symbolic–non-symbolic mapping process in children. A P2p-ERP component with similar right-parietal lateralization and timing as the current P2p gradually increased in amplitude as the novel symbols acquired numeric meaning by their mapping onto their non-symbolic counterparts. This effect did however only occur for novel symbols representing small magnitudes 1–4 that could be easily mapped onto corresponding non-symbolic representations via subitizing. In line with Dehaene et al.^26^, this led to the conclusion that the P2p reflects the (automatic) activation of magnitude information that has become associated with the novel symbols during learning. Second, multiple fMRI studies have found specific activation of right inferior parietal sulcus during number comparison tasks in which adults had to decide which of two symbolic (e.g., Arabic digits) or non-symbolic (arrays of dots) number stimuli represented a larger magnitude and concluded that this area houses an analog representation of magnitude and is involved in automatic or voluntary semantic processing of the magnitude dimension^27,39–41^. Whereas previous fMRI studies included standard symbolic or non-symbolic stimuli (Arabic digits or dot patterns), a link between right parietal activation and magnitude processing is also reported for finger-number stimuli in an fMRI study by Kaufmann et al.^42^. In this study, children and adults showed unique activation of right parietal areas during performance of a task in which they had to indicate which of two adjacently presented one-hand finger patterns showed the largest magnitude and not in a task with the same hand stimuli but now requiring the comparison of hand palm orientations (spatial comparison). Third and last, there are prior reports of canonical (cultural-specific) counting habits such as starting to count from 1 to 10 with the left or right hand, creating biases in adults’ performance on tasks that require processing of spatial-numerical relations, like positioning a number on a number line^43,44^.

Based on the findings from the prior brain studies discussed in the previous paragraph, we tentatively suggest that the current higher right-parietal P2p response to canonical finger montring postures showing numbers 2–4 reflects first activation of/or access to associated analog magnitude representations. One explanation for this P2p effect being only present for small one-hand finger patterns (2–4) and not for finger patterns 7–9, might be that finger-magnitude associations are established in the brain during the developmental pre-school phase in which children first use their fingers to acquire numerical representations^43^, which primarily involves small numbers 1–4. The semantic associations between canonical finger montring postures representing numbers 1–5 and their corresponding non-symbolic (magnitude) representation might be further strengthened by their more frequent use in daily communication. It is currently not clear why the same P2p response is not elicited by canonical patterns showing numbers large than 5, but this might be due to weaker direct associations with non-symbolic number representations, although this is speculative at this stage. It should be noted that small (2–4) and larger (7–9) canonical finger montring patterns facilitated math verification performance and enhanced P3 amplitudes to the same extent, the latter pointing to a special status of both in long-term memory. Although purely speculative, it might be that two-hand finger montring patterns that are not yet as frequently used in early childhood, have acquired stronger associations with other, later acquired symbolic representations of number than with non-symbolic representations. Last, although the above functional interpretation of the current P2p findings for canonical one-hand patterns fits with our and the reviewed data, the current study can however not confirm whether the current right lateralized parietal P2p data can be explained by a source in right parietal cortex and this thus needs confirmation in future studies.

In contrast to the P2p that was only enhanced in response to canonical finger patterns showing numbers smaller than 5, the later centro-parietal P3-ERP component did not show the predicted canonicity × numerical range interaction but instead showed a main effect of canonicity, with P3 amplitude being enhanced to both canonical montring patterns 2–4 and 7–9. The present main canonicity effect on the P3 resembles that reported by Soylu et al.^22^ in timing and location. These authors compared ERP responses elicited by canonical (montring and counting) and non-canonical finger-number stimuli showing numbers 1–4 and interpreted the enhanced P3 response to canonical finger patterns 1–4 as reflecting the retrieval of semantic, in this case numeric, information associated with the canonical finger-number patterns from memory. We extended these findings by also showing a P3 amplitude canonicity effect for canonical finger montring patterns 7–9 represented on two hands. We furthermore found a unique significant correlation between P3 amplitude and reaction time for canonical finger patterns showing correct sum solutions, with higher P3 amplitude being associated with faster sum verification times. But regarding the post-hoc and exploratory nature of the correlation analyses and the relatively small sample size of 28 adults, these correlation results should be carefully interpreted and need replication within a larger sample. Comparison of P3 results between Soylu’s et al.^22^ study and the present study is however complicated by differences in the tasks used and the moments in the task during which ERPs to the finger patterns were measured. Whereas in Soylu et al.^22^ ERPs were measured in response to finger patterns that preceded Arabic digits to which their numerosity had to be matched, in our task ERPs were measured in response to the finger patterns showing the solution to an earlier presented addition problem. In the present study, ERPs do thus not only reflect processes associated with the identification/extraction of the numerosity shown by the finger montring patterns but also reflect the processes involved in making the decision of whether the number indicated by the finger patterns matches with the solution of the prior presented addition problem or not (i.e., number comparison processes). In prior ERP studies using similar math verification paradigms but with sum solutions shown in the form of Arabic digits instead of finger montring patterns, similarly enlarged P3 amplitude on correct (versus incorrect) sum solutions trials has been consistently found and is linked to easier classification/categorization processes e.g., Refs.^36,45^, and/or higher intuitive level of response confidence^46,47^. Dickson and Wicha^36^ also found an effect of problem size with smaller math problems eliciting higher P3 responses, which they explained by smaller problems having gained stronger internalized representations and thus higher familiarity due to our more frequent encounters with small math problems in daily life. Interestingly, in addition to the main P3 canonicity effect on P3 amplitude, we also found an interaction between numerical range and sum solution. This interaction indicated that finger patterns conveying small numerosities 2–4 showed a larger correct/incorrect P3 increase than finger patterns 7–9. In line with the previous P3- and math verification ERP literature, we tentatively interpret the current P3 findings and their correlation with math verification time as signifying facilitated response decision/categorization processes of more familiar and easily recognizable canonical one-hand finger montring patterns, probably due to their stronger internalized representations in long-term memory.

In summary, cardinal canonical finger patterns of both numbers 2–4 and 7–9 facilitated young adults’ sum verification time when showing correct sum solutions. This behavioral facilitation was accompanied by an enhanced P3 response, which, based on prior literature, might be indicative of easier recognition/classification of cardinal canonical finger montring patterns due to their storage in long-term memory. Canonical finger montring patterns showing small numbers 2–4 in addition also facilitated performance on incorrect sum solution trials and elicited an early P2p response above right parietal cortex that was, based on prior brain studies, suggested to reflect the (automatic) activation of/access to associated analog representations of magnitude possibly originating from their frequent use in early childhood. Future longitudinal studies measuring children through different developmental phases should shed more light on when and how these cardinal finger patterns gain their canonical status, and how beneficial they are in the developmental phase in which children acquire the -symbolic number system.

## Methods

### Participants

Participants were thirty-four young adults recruited from the student population at Maastricht University, The Netherlands. One participant was excluded due to technical difficulties with the EEG recording, and five other participants were excluded because of below chance performance on the task. All reported analyses included the remaining 28 participants (mean age: 21 years 4 months [SD 1 years 6 months]; 2 left-handed; 8 males). All adults provided written informed consent after being informed about the study. Participants were rewarded with university course credits for their participation. The current study was approved by the local Ethical Review Committee Psychology and Neuroscience of Maastricht University, The Netherlands (ERCPN_RP2027_2019_34). All procedures were in accordance with the Declaration of Helsinki.

### Procedure

Testing took place at the dedicated EEG labs in a sound-proof booth and started with an explanation of the procedure, followed by attachment of the electrodes. Participants first performed another task, followed by the current Math verification task, and during both tasks their EEG was measured. Before the real math verification task started, all participants practiced the task until a performance criterion of 70–80% correct responses was reached to be sure that they understood the task instructions and were able to perform the task.

Instructions were to respond as fast and as accurately as possible, and participants were asked to minimize eye blinks and (eye) movements during the tasks.

### Math verification task

**Figure 5:**
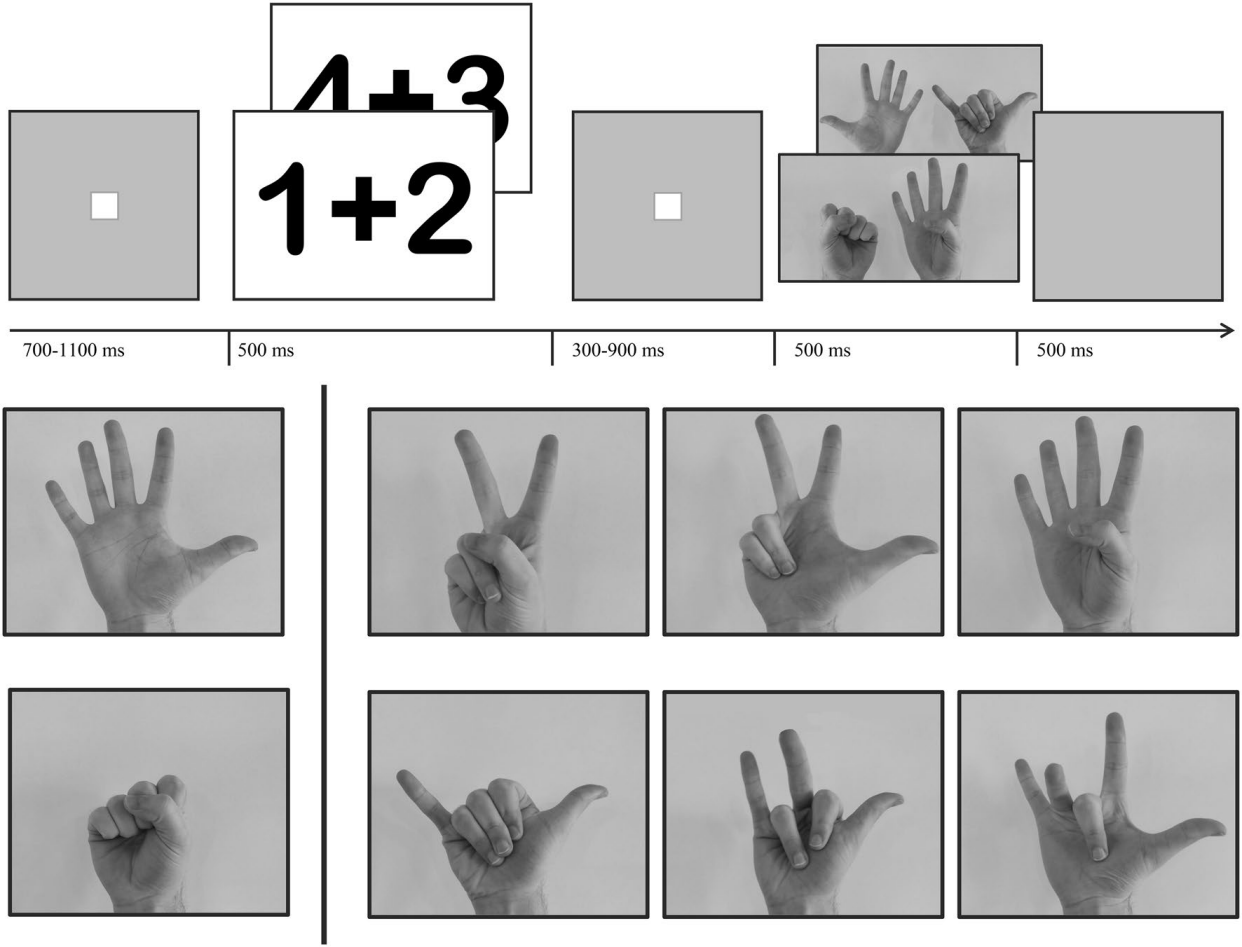
The Math Task (top) showing two examples of addition problems presented in Arabic Numerals, followed by a variable interval (between 300 and 900 ms), followed by a problem solution presented by the hands (foreground: incorrect problem solution represented by a canonical finger configuration; background: correct problem solution represented by a non-canonical finger representation). The montring finger stimuli (bottom) were composed of canonical (top row) or non-canonical (bottom row) patterns combined with an open (top row left) or closed hand (bottom row left). Task stimuli were drawn hands obtained from the internet (see Ref.^49^), but due to copyright considerations photos of the presented postures are shown in this figure.

In the math verification task, participants were presented with simple addition problems consisting of one-digit Arabic numerals (e.g., 2 + 4), followed by a solution shown in the form of canonical or non-canonical hand-finger configurations (see Fig. 5 for an example of a complete trial including timing information). The sums were chosen in such a way that sum solutions shown by the hands fell into the small (2–4) or large (7–9) number range. In 50% of the trials presented sum solutions were correct and in the other 50% incorrect and the participants’ task was to press the left button on a Cedrus RB-844 button box when the sum was correct and the right button in case it was incorrect.

The math verification task consisted of a total of 288 trials (presented in three blocks of 96 trials), of which half of the finger-number montring configurations represented an incorrect solution and the other half a correct solution. Further, sum solutions represented by the hands fell in either the small number range (2, 3, or 4) or the large number range (7, 8, or 9), and numerical range was balanced across the 288 trials. Across all trials, each solution was presented 24 times by non-canonical and 24 times by canonical hands. Stimulus presentation was quasi-random, with the restrictions of identical sum solutions never being presented directly after each other, and one type of hand stimulus (canonical/non-canonical) never shown more than four times in a row. In half of the incorrect trials the number represented by the hands had a distance of 1 from the correct Solution, while the other half had a distance of 2. All stimuli were presented on grey backgrounds inside white boxes at the center of a 19-inch monitor with a viewing distance of 57 cm using the PsychoPy python package^48^.

#### Selection of finger pattern stimuli

The selection of the non-canonical and canonical montring stimuli included in the present study was based on a small pilot questionnaire study with N = 14 students that were presented with a row of four pictures showing different finger patterns representing the same number. Two of the four presented patterns were different non-canonical patterns and the other two were the two possible canonical patterns with adjacently raised fingers but one canonical pattern including the thumb (often called ‘counting’ pattern) and the other canonical pattern without the thumb (often called ‘montring’ pattern). The students were asked to rate the four finger patterns on the likelihood that they would use them to communicate a number to someone else (scale 1–4, with 1 highest likelihood and 4 lowest likelihood). For each of the included numbers 2, 3, and 4, out of the two non-canonical finger patterns we chose the pattern that was rated as most unlikely being used for communicating the number. Of the two canonical patterns we (for each number) chose the canonical pattern that was indicated by the majority as the most likely used way to communicate the number (although both canonical patterns did not differ much in rating but both differed strongly in rating from both non-canonical patterns). This resulted in the non-canonical and canonical finger patterns shown in Fig. 5 (note that the same finger patterns for 2, 3 and 4 were used for higher numbers 7, 8, 9, only accompanied by a full-hand indicating 5, whereas in the case of 2, 3, 4 a second hand was also shown but with a closed fist representing zero).

### EEG/ERP acquisition and analyses

Electro-Encephalographic (EEG) measurements took place in a lab at the University. EEG data were recorded using an elastic EEG-electrode cap (EasyCap; Nellcor-Puritan Bennet, Hayward, CA) with a 38 tin electrode set-up (FP1, FP2, F7, F3, Fz, F4, F8, FC5, FC1, FCZ, FC2, FC6, T7, C3, CZ, C4, T8, CP5, CP1, CPZ, CP2, CP6, P7, P3, PZ, P4, P8, PO7, O1, OZ, O2, A2, AFZ, A1). The data were filtered online at 0.01–225 Hz and continuously sampled at a rate of 500 Hz using a BrainAmp amplifier system and software (BrainVision Analyzer, Vers. 2.2.0, Brain Products GmbH, Gilching, Germany^50^). The AFz electrode served as ground, and the left mastoid (A1) acted as the online reference; the right mastoid (A2) was included as an extra active electrode. Horizontal and vertical EOG was measured by electrodes placed on respectively the outer canthus of each eye and above/below the left orbit. All electrode impedances were kept below 5–10 kOhm and were frequently checked during testing.

For offline analyses of the EEG data, Matlab2019a/EEGlab2019/ERPLABv7.0.0 software was used^38,51,52^. The data was first resampled to 250 Hz and re-referenced to the average signal. Next, a band-pass filter of 0.1–70 Hz was applied to the data before execution of Independent Component Analysis (ICA) for removal of horizontal eye movements and blinks. The ocular artifact-free data was filtered using a 30 Hz Low-pass filter, after which the data was epoched (based on the dot-quantity and symbol-quantity event-codes, excluding epochs with incorrect responses) into 300 ms pre-stimulus and 1000 ms post-stimulus windows. Baseline correction was performed using the pre-stimulus interval. Next, an automatic artifact detection procedure was applied to the remaining epochs (only at electrodes P3, P4, and the average channel of Cz and CPz that were respectively included in the P2p and P3 analyses), rejecting trials with activity exceeding a ± 50 µV. Participants were only included in the subsequent analyses if at least 50% of total trials remained for averaging (after removal of incorrectly responded and artifact trials). This led to the removal of three participants based on accuracy and two participants based on EEG artifacts, as mentioned in the participant section. Of the remaining 28 participants, an average of 27.13% (27.21% [9.72–44.44%] due to incorrect responses, and 1.17% [0–18.97%] due to artifacts) of the total number of trials were rejected.

The choice of electrodes and time-windows for the P2p and P3 analyses was based on (1) the previous number processing ERP literature (see “Introduction”) in which similar number processing (comparison) tasks and/or similar finger stimuli were used and (2) on visual inspection of the grand average ERP signals and topography maps in the different conditions (see Figs. 2 and 3 for grand average ERPs and topo-maps). This led to the analyses being focused on an early positivity (P2p) in an early 220–310 ms time window at left and right parietal (P3 and P4^13,26,27,29–31,53^) electrodes, and a later positivity (P3) in a 280–550 ms window at CPz and Cz electrodes^22,26,28,29,32–34^. Mean amplitude values within these time windows were entered in the statistical analyses.

### Statistical analysis

For dependent measures reaction time (RT), P2p amplitude and P3 amplitude, Linear Mixed-effects models were constructed in IBM SPSS 27^54^. For the accuracy data, Generalized Estimation Equation (GEE) models were constructed because of severe violation of normality. All models included within-subject factors Canonicity (2 levels: canonical and non-canonical), sum Solution (2 levels: correct and incorrect sum solution trials), and numerical Range (2 levels: low numerical range [numbers 2, 3, 4] and high numerical range [numbers 7, 8, 9]). The P2p amplitude analysis included an extra factor Hemisphere (2 levels: left and right).

Assumptions were checked using a compound Symmetry covariance structure, removing single observations (single data-points) as outliers if residuals exceeded three standard deviations. No observations were excluded for the ERP analysis. For the behavioral data, a single outlier was removed for RT, while six observations were removed from the accuracy analysis. Assumptions of normality and homoscedasticity were met in all analyses. For dependent variables RT and ERP (P2p and P3) amplitude, various covariance matrices were compared using AIC to determine the best fitting model. For the RT and P2p-ERP models, the Toeplitz structure showed the best and more parsimonious fit, whereas the P3 data fitted best with a Compound Symmetry structure. All models were subsequently reduced by removing non-significant four-way and three-way interactions. Non-significant two-way interactions were purposely kept in the model; removing them did not change any of the conclusions.

## Data Availability

The data files containing the raw behavioral data and the SPSS matrix containing the behavioral and ERP data used in the presented analyses will be available via the DataverseNL repository, https://doi.org/10.34894/4OEBD9. The raw EEG-data will be available upon reasonable request by sending an e-mail to l.jonkman@maastrichtuniversity.nl.
